# Out-of-hospital cardiac arrest in Norwegians aged 12–49 years: nationwide analysis of preceding symptoms and risk factors related to aetiology and pre-arrest exercise habits

**DOI:** 10.1136/bmjsem-2025-002505

**Published:** 2025-07-01

**Authors:** Cecilie Benedicte Isern, Roald Bahr, Malin Flønes, Harald Jorstad, Jo Kramer-Johansen, Ingrid Mjøs, Arne Stray-Pedersen, Hilde Moseby Berge

**Affiliations:** 1 University of Oslo Faculty of Medicine, Oslo, Norway; 2 Prehospital Division, Oslo University Hospital, Oslo, Norway; 3 Department of Sports Medicine, Norwegian School of Sports Sciences, Oslo, Norway; 4 Aspetar Orthopaedic and Sports Medicine Hospital, Doha, Qatar; 5 Cardiology, Amsterdam University Medical Centres, Amsterdam, The Netherlands; 6 Division of Prehospital Services, Oslo University Hospital, Oslo, Norway; 7 Institute of Clinical Medicine, University of Oslo Faculty of Medicine, Oslo, Norway; 8 Department of Forensic Sciences, University of Oslo Faculty of Medicine, Oslo, Norway; 9 Oslo Sports Trauma Research Center, Department of Sports Medicine, Norwegian School of Sports Sciences, Oslo, Norway; 10 Department of General Practice, Institute of Health and Society, University of Oslo Faculty of Medicine, Oslo, Norway

**Keywords:** exercise, cardiovascular epidemiology, cardiology prevention

## Abstract

**Background:**

Out-of-hospital cardiac arrest (OHCA) in the young is a tragic event. Regular exercise reduces cardiovascular disease (CVD) risk but, at certain intensities and volumes, is associated with increased OHCA risk. Understanding symptoms, risk factors and aetiology is central for primary prevention.

**Objective:**

Assess symptoms and risk factors related to OHCA aetiology in young Norwegians, and the role of exercise volume.

**Method:**

We obtained data from the Norwegian Cardiac Arrest Registry (2015–2017), medical records, autopsy reports and questionnaires. Inclusion criteria were ages 12–49 years and OHCA of presumed cardiac aetiology.

**Results:**

Data from 134 individuals (81 survivors, 53 deceased) showed that CVD symptoms were present during both rest/everyday activity and exercise in most victims, and were reported by more survivors than by next-of-kin of the deceased (78% vs 60%). Only 12% had symptoms just during exercise. Ischaemic heart disease was the leading cause (58% in males vs 38% in females), followed by structural, non-ischaemic heart disease. Sudden unexpected death syndrome (SUDS) was most common in individuals aged ≤35 years. Risk factors were present in 74%, with family history for CVD most prevalent (51%) and overweight in at least 33%. There were no significant differences in symptoms, risk factors or OHCA aetiology related to exercise volume prior to OHCA.

**Conclusion:**

Symptoms and CVD risk factors were prevalent in young Norwegians suffering OHCA regardless of exercise volume. Ischaemic heart disease was the leading cause of OHCA. We suggest evaluating symptoms carefully and addressing risk factors to prevent OHCA in young Norwegians regardless of exercise habits.

**WHAT IS ALREADY KNOWN ON THIS TOPIC**

- Exercise benefits cardiovascular health but may also trigger out-of-hospital cardiac arrest (OHCA).
- Symptoms during exercise are common among young OHCA victims, but whether they have symptoms also during rest/everyday activity has not been explored.
- Aetiology of OHCA varies between countries.

**WHAT THIS STUDY ADDS**

- Cardiovascular risk factors and symptoms were prevalent regardless of exercise volume prior to OHCA, with symptoms reported during both rest/everyday activities and exercise.
- Ischaemic heart disease was the leading cause of OHCA in both genders, and sudden unexplained death syndrome (SUDS) was most common in those aged ≤35 years.
- Genetic testing was underused in SUDS cases.

**HOW THIS STUDY MIGHT AFFECT RESEARCH, PRACTICE OR POLICY**

- Cardiovascular symptoms may be more common than previously reported, necessitating thorough assessment of symptoms both during exercise and rest/everyday activities, regardless of exercise habits.
- Improved diagnosis and hence prevention of OHCA require increased genetic testing and assessments in larger hospitals following national autopsy guidelines.

## Introduction

 Out-of-hospital cardiac arrest (OHCA) in the young is a tragic event with high mortality rates and considerable social impact. Regular exercise generally reduces the risk of cardiovascular disease (CVD) but, at certain intensities and volumes, is associated with an increased risk of CVD events.^1 2^

To reduce OHCA mortality in the young, knowing the aetiology and understanding the symptoms and risk factors preceding these events is important. Recent evidence suggests that symptoms and risk factors of CVD are common among young OHCA victims, including athletes.^3 4^ Currently, systematic preventive measures are recommended only for participants in competitive sports.^5^,^7^ Except for one study,^8^ existing data on symptoms and risk factors prior to OHCA are limited to deceased victims, underusing valuable insights from survivors. Furthermore, there is a call for more data related to OHCA in females.^9^ To our knowledge, no study has explored the relationship between exercise volume and the symptoms or risk factors preceding OHCA. Investigating these factors is crucial for understanding the potential role of exercise volume in risk assessment prior to OHCA. Therefore, through a multiple source approach including both survivors and deceased of both genders, the main aim of this study was to assess preceding symptoms and risk factors related to OHCA aetiology in young Norwegians, and the role of exercise volume.

## Methods

We performed a retrospective observational study to assess preceding symptoms and risk factors in young Norwegians with OHCA.

### Patient and public involvement

User representatives participated in the planning of the study. The group included one adult OHCA survivor and their next-of-kin, the next-of-kin of a deceased adult, a youth OHCA survivor and a deceased youth. All user representatives had experiences from sports-related OHCA. They contributed by sharing their experiences in an interview, as well as providing feedback to the study invitation and the questionnaire. We made formal changes to the study invitations and added additional questions and explanatory text to the questionnaire based on the feedback.

### Equity, diversity and inclusion statement

We included patients regardless of ethnicity and gender. All patients registered in NorCAR aged 12–49 years at the time of OHCA were invited to participate. For practical reasons, we only included patients with a Norwegian birth identification number. To ensure broader inclusion, we distributed the study invitation via postal services. Data on aetiology were analysed by gender. The study group consisted of 50% females, with representatives from various research fields at different stages of their research careers.

### Study design, study population and data collection

**Figure 1:**
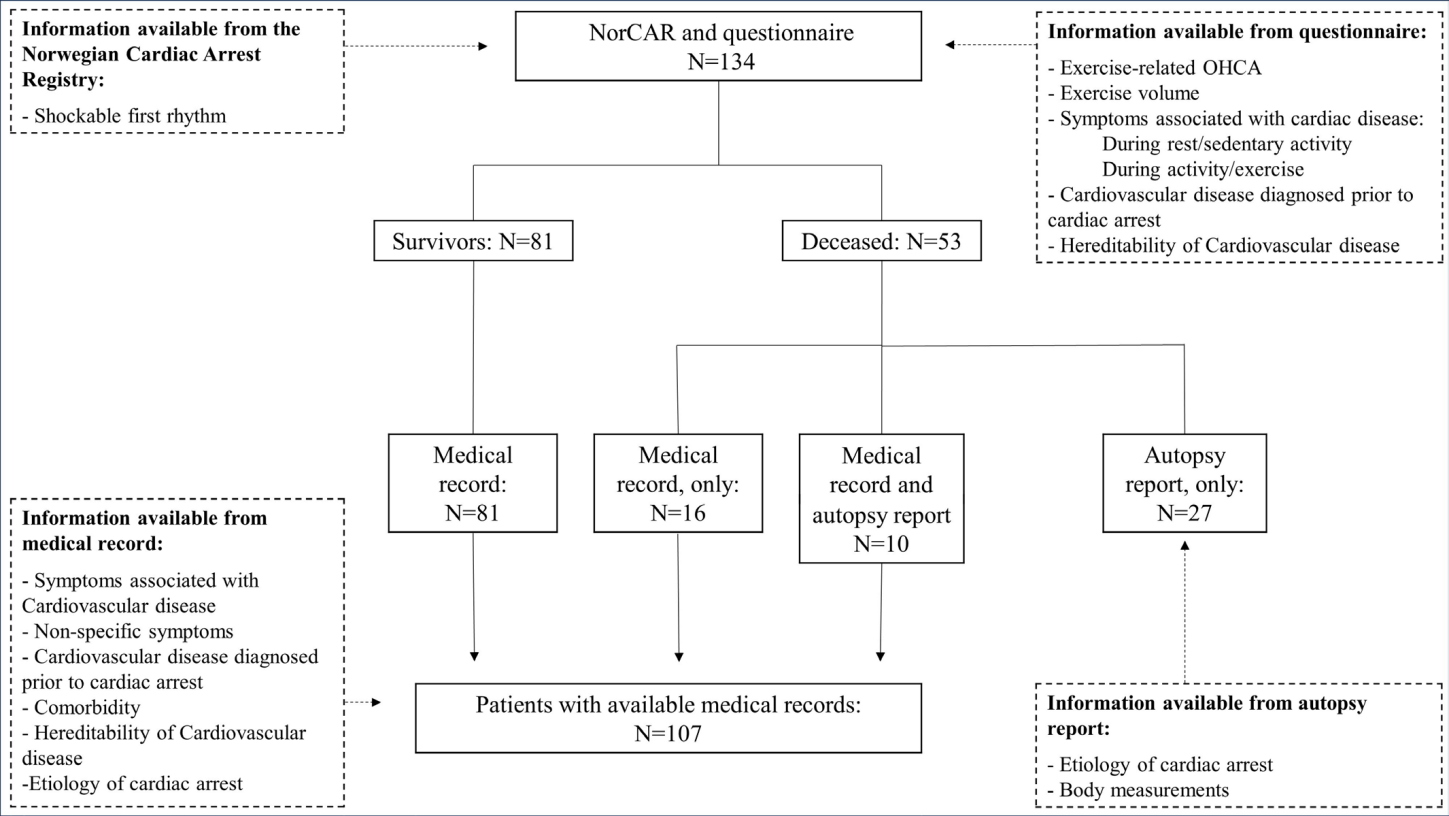
Flow chart illustrating the data sources and respective data variables. NorCAR, Norwegian Cardiac Arrest Registry; OHCA, out-of-hospital cardiac arrest.

The methods have been described extensively elsewhere.^10^ In short, we used a multisource approach to collect data (figure 1). NorCAR was the primary data source. A questionnaire developed for the study provided additional data from survivors or next-of-kin of the diseased. We retrieved medical records and autopsy reports when applicable.

We included Norwegian citizens aged 12–49 years with OHCA from 1 January 2015 through 31 December 2017. Additional criteria for inclusion from NorCAR were presumed cause of OHCA registered as cardiac, respiratory failure or drowning. The category ‘presumed cardiac cause’ in NorCAR includes all patients with no other obvious cause of OHCA. Survivors or next-of-kin of the deceased eligible for inclusion with a valid postal address in the Norwegian Population Register received a study invitation and a questionnaire by mail. We requested medical records after consent, in addition to autopsy reports for the deceased. We excluded patients with insufficient information to determine OHCA aetiology, such as those lacking medical records or autopsy reports, as well as patients with confirmed non-cardiac aetiology (such as respiratory failure or drowning accident).

### Data sources

#### Norwegian Cardiac Arrest Registry

Cardiac arrest is a mandatory reportable condition in Norway from 2013, regulated by Norwegian law.^11^ We included cases from the first 3 years of near complete data collection to the registry.^12^ The criterion for registration is cardiopulmonary resuscitation performed on an unresponsive individual by bystanders, first responders or healthcare personnel.

#### Questionnaire

The first and last authors developed the questionnaire to collect information in line with the recommendations for preparticipation screening of athletes by the Sports Cardiology Section of the European Association for Cardiovascular Prevention and Rehabilitation.^13^ Patient representatives contributed to the development, but no formal validation was performed. The questionnaire included 34 multiple-choice questions and 22 free-text answers (online supplemental table S1). Relevant for this paper, we gathered data on CVD diagnosed prior to OHCA, and CVD preceding symptoms (chest pain, dyspnoea, palpitations, syncope or near-syncope). We noted whether the symptoms occurred during rest/daily life activity and/or exercise and asked about healthcare consultations prompted by these symptoms. Data from family history was considered positive if arterial hypertension, hypercholesterolaemia, diabetes mellitus, cardiac disease or sudden unexpected death was reported in family members before 50 years of age. Participants reported average weekly exercise hours in predefined categories (0 hours/week, <5 hours/week or ≥5 hours/week), referred to as pre-OHCA exercise volume in this paper.

#### Medical records

We obtained medical records from primary and specialist care from 3 years prior to the incident. Data extraction followed a predefined protocol encompassing aetiology of OHCA, CVD and non-CVD comorbidities, symptoms prior to OHCA (both associated with CVD and non-specific), CVD risk factors (overweight, arterial hypertension, hypercholesterolaemia, diabetes mellitus), as well as family history of CVD or OHCA and related risk before 50 years of age.

#### Autopsy reports

The police regularly request a medicolegal examination (forensic autopsy) in the event of sudden unexpected death. These reports were obtained from the police districts with permission from the Director of Public Prosecutions. We recorded information from the autopsy reports in predefined categories: OHCA circumstances, available medical history, medication, toxicology analyses from blood, eye liquid and urine, external findings including body weight and height, macroscopic findings including heart weight, microscopic findings and aetiology of OHCA.

We also had access to medical autopsy reports if they were attached to the medical record from the hospital.

### Adjudicated aetiology

The first and last author reviewed available medical documentation and autopsy reports to determine OHCA aetiology. We discussed ambiguous patients in plenary with all authors. A specialist in forensic medicine reviewed all patients with available autopsy reports and provided conclusions on the aetiology of death. Information from autopsy reports was considered superior to medical records regarding the aetiology of OHCA. If no cause of OHCA could be determined, the aetiology was classified as sudden unexplained death syndrome (SUDS). For analysis, we categorised aetiology into four groups: ischaemic heart disease (IHD), structural non-IHD, primary electrical disturbances and SUDS.^14^

### Data definitions

We defined our study population as ‘young’ since all participants were younger than the median age of general OHCA populations.^11 15 16^ Patients who reported no regular exercise prior to OHCA were classified as non-regular exercisers, while regular exercisers were categorised into two groups: <5 hours/week and ≥5 hours/week. Data on exercise volume were reported for the last year prior to OHCA. We defined exercise-related OHCA as OHCA occurring during exercise or within 1 hour after exercise.^10^ Patients were categorised as overweight if they had a body mass index (BMI) ≥25 or if the body build was described as above normal, overweight or other similar expressions. Patients whose medical records lacked descriptive data on body build were classified as having normal weight. We considered the following five non-specific symptoms as angina equivalent: general exhaustion, dizziness, reduced physical capacity, nausea and stomach pain.

### Statistical methods

Data are presented for the overall study population, and in groups divided by pre-OHCA exercise volume, age or gender. Data were analysed using the statistical software package IBM SPSS Statistics for Windows (V.26.0; IBM, Armonk, New York, released 2019). Non-normally distributed variables are presented as median and IQR. Hypothesis testing for non-normally distributed variables was performed by non-parametric testing. We used the χ^2^ test to compare categorical variables, or Fisher’s exact test if expected count was <5. All tests were two-tailed and p<0.05 was considered statistically significant. In addition to p value, hypothesis testing results are presented by effect size (ES) calculated by Cramer’s V. For outcomes with 1 df, an ES <0.3 indicates a small effect, 0.3<ES<0.50 is considered moderate and ES >0.50 indicates a large effect.^17^ For outcomes with 2 df, ES ≤0.20 is considered small, 0.21<ES<0.35 as moderate and ES >0.35 as large. Hypothesis testing by χ² additionally includes the χ² and df. If statistical significance was obtained for variables with ≥3 categories, we performed post hoc analysis using pairwise comparison to determine p values for differences between the groups. *To avoid increased rates of type I errors in* these cases, we used Bonferroni correction to adjust the significance level for multiple testing.

## Results

### Study population

During the 3-year study period, 588 patients were eligible for inclusion from NorCAR and available for a study invitation (online supplemental figure S1).^10^ Among these, 236 (40%) consented to participate. We excluded 55 patients who did not have sufficient information to adjudicate OHCA aetiology and 47 patients with confirmed non-cardiac aetiology. The final study population comprised 134 patients; 81 (60%) survivors and 53 deceased (figure 1, table 1). Their median age was 43.4 years (range 36–47) and included 37 women (28%). Data on pre-OHCA exercise volume were available for 124 (93%) of the 134 patients: one-quarter reported no regular exercise, 60 (48%) exercised 1–4 hours per week and 34 (27%) exercised ≥5 hours per week. The median time from OHCA to questionnaire completion was 2.2 years (IQR 1.8–2.8).

**Table 1:** Descriptive statistics of the study population, overall and grouped by pre-OHCA exercise volume

	Overall study population	No regular exercise	<5 hours of exercise per week	≥5 hours of exercise per week	Test used	χ² value (df)	P value	Effect size
	N (%)	N (%)	N (%)	N (%)				
Source of data	Norwegian Cardiac Arrest Registry and questionnaire							
	*134*	*30*	*60*	*34*				
Age, median (IQR)	43.4 (11.1)	45.0 (5.9)	43.0 (13.3)	42.5 (14.0)			0.181	
Age ≤35 years	34 (25)	5 (17)	16 (27)	11 (32)	χ²	2.09 (2)	0.384	0.27
Gender, female	37 (28)	11 (37)	17 (28)	8 (24)	χ²	1.36 (2)	0.506	0.11
First rhythm shockable*	86 (64)	18 (67)	38 (72)	24 (73)	χ²	0.30 (2)	0.859	0.05
Exercise-related OHCA	24 (18)	2 (6.7)	11 (18)	10 (29)	χ²	5.46 (2)	0.065	0.21
Survivors at 30 days post-OHCA	81 (60)	15 (50)	39 (65)	22 (65)	χ²	2.13 (2)	0.345	0.13
Source of data	Questionnaire and medical record if available							
	*134*	*30*	*60*	*34*				
**Pre-existing CVD**					FET	N/A	0.901	0.12
Ischaemic heart disease	3 (2.2)	1 (3.3)	1 (1.7)	1 (2.9)				
Structural non-ischaemic heart disease	2 (1.5)	1 (3.3)	1 (1.7)	0				
Primary electrical CVD	6 (4.5)	0	4 (6.7)	2 (5.9)				
Multiple CVD	4 (3.0)	1 (3.3)	2 (3.3)	1 (2.9)				
No CVD	119 (89)	27 (90)	52 (87)	30 (88)				
**Risk factors for CVD**								
Positive family history: CVD or OHCA	48 (36)	13 (43)	19 (32)	15 (44)	χ²	1.93 (2)	0.382	0.13
Positive family history: HTA, hypercholesterolaemia or diabetes mellitus	43 (32)	10 (33)	21 (35)	11 (32)	χ²	0.07 (2)	0.964	0.02
Negative family history	65 (49)	14 (47)	27 (45)	16 (47)	χ²	0.05 (2)	0.978	0.02
Source of data	Medical record and/or autopsy report							
	*134*	*30*	*60*	*34*				
Overweight	46 (34)	13 (43)	19 (32)	9 (26)	χ²	2.15 (2)	0.341	0.13
Source of data	Medical record, only							
	107	22	50	28				
HTA	15 (11)	5 (23)	5 (10)	4 (14)	FET	N/A	0.336	0.14
Hypercholesterolaemia	15 (11)	3 (14)	10 (20)	2 (7.1)	FET	N/A	0.377	0.15
Diabetes mellitus	12 (11)	*6 (27)*	*6 (12)*	*0*	FET	N/A	0.009	0.30
No CVD or risk factors for CVD, excluding family history	52 (49)	8 (36)	22 (44)	18 (64)	χ²	4.49 (2)	0.106	0.21
No CVD or risk factors for CVD, including family history	28 (26)	4 (18)	11 (22)	10 (36)	χ²	2.50 (2)	0.287	0.16
**Comorbidity, excluding CVD**								
Autoimmune disease	14 (13)	6 (27)	6 (12)	2 (7.1)	FET	N/A	0.142	0.21
Pulmonary disease	8 (7.5)	0	3 (6.0)	5 (18)	FET	N/A	0.063	0.24
Neurological disease	4 (3.7)	3 (14)	1 (2.0)	0	FET	N/A	0.056	0.27
Other	34 (32)	9 (41)	16 (32)	6 (21)	χ²	2.23 (2)	0.328	0.15
No comorbidities, excluding CVD	57 (53)	9 (41)	28 (56)	16 (57)	χ²	1.67 (2)	0.435	0.13
Previously healthy with no CVD, no risk factors for CVD or other comorbidities	16 (15)	2 (9.1)	7 (14)	7 (25)	FET	N/A	0.327	0.16

Numbers are presented as n (%), except for age. Statistically signifcant results are presented in italics.

* The nominator include cases that were successfully defibrillated before arrival of EMS, cases that for other reasons had circulation at arrival of EMS are counted as missing. Hypothesis testing of between-group differences is calculated by FET if any expected value is <5. Effect size is presented by Cramer’s V.

CVD, cardiovascular disease; EMS, Emergency Medical Services; FET, Fisher’s exact test; HTA, arterial hypertension; N/A, not applicable; OHCA, out-of-hospital cardiac arrest; χ², chi-square test.

### Aetiology of OHCA

**Figure 2:**
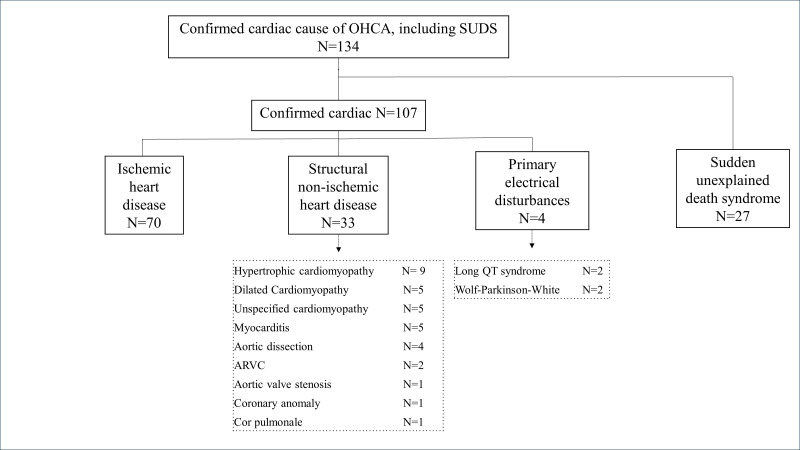
Overview of the adjudicated aetiology of the OHCA, reported for cases with available medical record and/or autopsy report. ARVC, arrhythmogenic right ventricular cardiomyopathy; OHCA, out-of-hospital cardiac arrest; SUDS, sudden unexpected death syndrome.

Of the 134 patients, 107 had an identifiable cardiac aetiology (figure 2); 70 (52%) had IHD, followed by structural non-IHD (n=33), SUDS (n=27) and primary electrical disturbances (heart rhythm disorder) (n=4). Hypertrophic cardiomyopathy (HCM) was the most prevalent subgroup within the structural non-ischaemic group (9/33).

**Figure 3:**
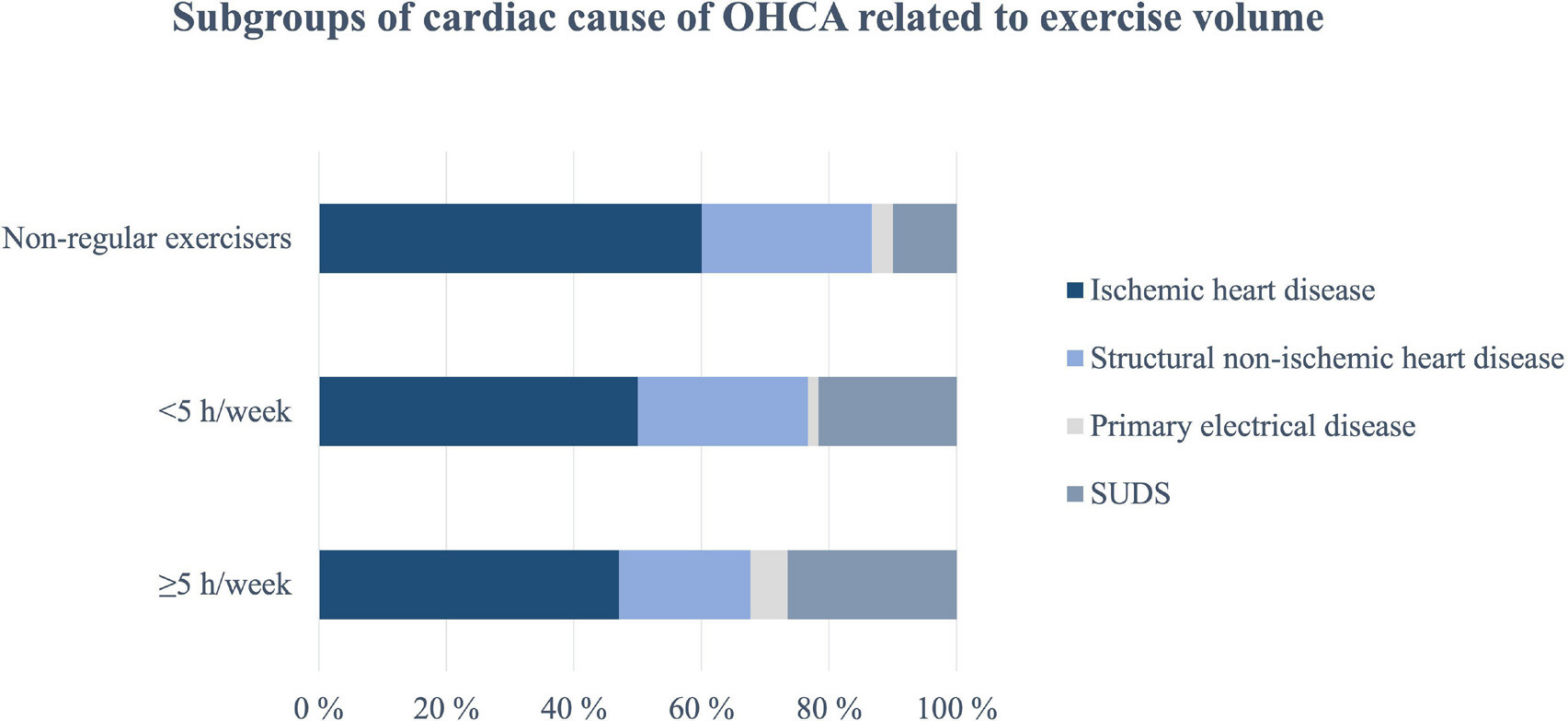
Overview of the adjudicated aetiology of the OHCA presented by groups of pre-OHCA exercise volume, reported for cases with available medical record and/or autopsy report. OHCA, out-of-hospital cardiac arrest; SUDS, sudden unexpected death syndrome.

IHD was the most common cause of OHCA independent of exercise volume and gender (figure 3, online supplemental table S2 and online supplemental figure S2) and was also the dominant cause in respondents aged >35 years (63%) (online supplemental figure S3a). SUDS (35%) was most common in patients aged ≤35 years (online supplemental figure S3b).

### Symptoms related to circumstances, exercise volume and gender

CVD symptoms were more commonly reported by survivors than by next-of-kin of the deceased (table 2). Less than one-quarter of the survivors (n=18, 22%) had no history of CVD symptoms prior to OHCA. Half of the survivors (n=42, 52%) reported at least two symptoms and nearly 10% (n=8) had experienced all CVD symptoms. The majority (n=28, 60%) of the 47 survivors who reported CVD symptoms in the questionnaires had experienced symptoms both at rest/during daily activity and during exercise (table 3), while 13 (28%) had symptoms only at rest/during daily activity. Experiencing symptoms only at rest was most common among non-regular exercisers (n=4, 50%) but it was also reported by 28% (n=7) of regular exercisers who exercised <5 hours/week and by 17% (n=2) of high-volume exercisers (online supplemental tables S4 and S5). Among the total 73 respondents (including both survivors and next-of-kin of the deceased) who reported CVD symptoms in the questionnaire (table 3), 36 (49%) reported that the patient had sought medical attention.

**Table 2:** Symptoms experienced prior to OHCA as retrospectively reported in questionnaire or medical record, and as described in medical records preceding the event

	Overall study population	Ischaemic heart disease	Structural non-ischaemic heart disease	Primary electrical disease	Sudden unexpected death syndrome	Test used	χ² value (df)	P value	Effect size
	N (%)	N (%)	N (%)	N (%)	N (%)				
**(a) Survivors**									
Survivors at risk	81	47	14	3	17				
Symptoms									
Chest pain	43 (53)	31 (66)	7 (50)	3 (100)	2 (12)	FET	N/A	<0.001	0.47
Dyspnoea	40 (49)	26 (55)	8 (57)	3 (100)	3 (18)	FET	N/A	0.008	0.37
Palpitations	29 (36)	13 (28)	6 (43)	3 (100)	7 (41)	FET	N/A	0.067	0.30
Near syncope or syncope	23 (28)	12 (26)	4 (29)	1 (33)	6 (35)	FET	N/A	0.859	0.09
Number of symptoms									
No symptoms reported	18 (22)	10 (21)	2 (14)	0	6 (35)	FET	N/A	0.239	0.26
One	21 (26)	11 (23)	5 (36)	0	5 (29)				
Two	20 (25)	13 (28)	2 (14)	0	5 (29)				
Three	14 (17)	7 (15)	4 (29)	2 (67)	1 (5.9)				
Four	8 (9.9)	6 (13)	1 (7.1)	1 (33)	0				
**(b) Deceased**									
Deceased at risk	53	23	19	1	10				
Symptoms									
Chest pain	15 (28)	13 (57)	1 (5.3)	0	1 (10)	FET	N/A	<0.001	0.47
Dyspnoea	15 (28)	7 (30)	6 (32)	0	2 (20)	FET	N/A	0.008	0.37
Palpitations	9 (17.0)	1 (4.3)	5 (26)	0	3 (30)	FET	N/A	0.067	0.30
Near syncope or syncope	14 (26)	4 (17)	5 (26)	1 (100)	4 (40)	FET	N/A	0.859	0.09
Number of symptoms									
No symptoms reported	21 (40)	6 (26)	10 (53)	0	5 (50)	FET	N/A	0.239	0.26
One	18 (34)	11 (48)	4 (21)	1 (100)	2 (20)				
Two	9 (17)	4 (17)	3 (16)	0	2 (20)				
Three	3 (5.7)	2 (8.7)	1 (5.3)	0	0				
Four	2 (3.8)	0	1 (5.3)	0	1 (10)				

Symptoms associated with cardiac disease is presented for the overall study population (n=134) and the subgroups of cardiovascular aetiology. Table 2(a) presents data for survivors and table 2(b) presents data for the deceased.

Hypothesis testing of between-group differences is calculated by FET if any expected value is <5. Effect size is presented by Cramer’s V.

FET, Fisher’s exact test; N/A, not applicable; OHCA, out-of-hospital cardiac arrest; χ², chi-square test.

**Table 3:** Detailed information reported by survivors and by next-of-kin on behalf of deceased on whether the symptoms prior to the event had been experienced: (I) only during rest/everyday activity, (II) during exercise, (III) both during rest/everyday activity and during exercise only

Source of data: questionnaire	Circumstances of symptoms reported by survivors					
	Number of persons with symptoms	Total number of symptoms reported	Chest pain	Dyspnoea	Palpitations	Near syncope or syncope
	N (%)	N (%)	N (%)	N (%)	N (%)	N (%)
**(a) Survivors**						
Patients who reported circumstances of the symptom of interest	47	90	20	26	26	18
I: Rest/Everyday activity, only	13 (28)	28 (31)	5 (25)	6 (23)	10 (39)	7 (39)
II: Exercise, only	6 (13)	11 (12)	3 (15)	2 (7.7)	3 (12)	3 (17)
III: Both; rest/everyday activity and exercise	28 (60)	51 (57)	12 (60)	18 (69)	13 (50)	8 (44)
**(b) Deceased**						
Patients who reported circumstances of the symptom of interest	26	43	8	13	9	13
I: Rest/Everyday activity, only	14 (54)	24 (56)	5 (63)	5 (38)	6 (67)	8 (62)
II: Exercise, only	3 (12)	5 (12)	1 (13)	3 (23)	0	1 (7.7)
III: Both; rest/everyday activity and exercise	9 (35)	14 (33)	2 (25)	5 (38)	3 (33)	4 (31)

The denominator in this equation were the number of respondents who reported the symptom of interest and the requested information about the circumstances related to activity. The information is presented separately for the overall study population of (a) survivors (b) deceased.

Non-specific symptoms were reported in 43 (53%) of the overall 81 patients with a history of CVD symptoms and available medical record. The most described non-specific symptoms were general exhaustion (22%), dizziness (16%) and pain in the shoulder, neck or back (14%) (table 4). Only one in five reported non-specific symptoms alone. Among patients with accessible medical records, only 11% (n=9) of the survivors and 23% (n=6) of next-of-kin of the deceased reported neither CVD-related nor non-specific symptoms.

**Table 4:** Presentation of non-specific symptoms extracted from the medical records for the overall study population, and for the subgroups of cardiac aetiology

	Medical record, only					P value	Effect size
	Overall study population	Ischaemic heart disease	Structural non-ischaemic heart disease	Primary electrical disease	Sudden unexpected death syndrome		
	N (%)	N (%)	N (%)	N (%)	N (%)		
Survivors, number of patients	107	62	21	3	21		
Non-specific symptoms							
Generally exhausted*	24 (22)	12 (19)	8 (38)	0	4 (19)	0.287	0.20
Dizziness*	17 (16)	7 (11)	8 (38)	1 (33)	1 (4.8)	0.012	0.33
Reduced physical capacity*	7 (6.5)	4 (6.5)	3 (14)	0	0	0.354	0.19
Nausea*	6 (5.6)	2 (3.2)	4 (19)	0	0	0.054	0.29
Stomach pain*	4 (3.7)	2 (3.2)	1 (4.8)	1 (33)	0	0.163	0.28
Shoulder/Neck/Back pain	15 (14)	13 (21)	2 (9.5)	0	0	0.083	0.25
Paraesthesia/Weakness in arms	6 (5.6)	6 (9.7)	0	0	0	0.353	0.21
Jaw pain	2 (1.9)	2 (3.2)	0	0	0	1.000	0.12
Symptoms associated with infectious disease in the last days	8 (7.5)	3 (4.8)	2 (9.5)	1 (33)	2 (9.5)	0.239	0.19
Any angina equivalent symptom	38 (36)	20 (32)	12 (57)	1 (33)	5 (24)	0.111	0.23
Number of angina equivalent symptoms						0.016	0.26
None reported	69 (64)	42 (68)	9 (43)	2 (67)	16 (76)		
One	22 (21)	14 (23)	3 (14)	0	5 (24)		
Two	12 (11)	5 (8.1)	6 (29)	1 (33)	0		
Three	4 (3.7)	1 (1.6)	3 (14)	0	0		

The table present the prevalence of each reported symptom, as well as the overall prevalence of any angina equivalent symptom, and the number of angina equivalent symptoms.

* Angina equivalent symptoms. Hypothesis testing of between-group differences is calculated by FET for as expected value is <5 for all tests. Effect size is presented by Cramer’s V.

We did not find any significant differences in the type and number of CVD symptoms (online supplemental table S3), non-specific symptoms (online supplemental table S6) and circumstances of CVD symptoms (online supplemental tables S4 and S5) when comparing the non-regular and regular exercisers. Palpitations were more common in female survivors (52%) compared with male survivors (30%) (χ² =value: 3.39, df=1, p=0.07, ES=0.21), but none of the differences in type or number of symptoms related to gender were statistically significant (online supplemental table S7).

### Symptoms related to aetiology of OHCA

Chest pain and dyspnoea were significantly more prevalent in IHD compared with SUDS (χ²=14.7, df=1, p<0.001 and ES=0.48/dyspnoea: χ²=7.15, df=1, p=0.007 and ES=0.33 for chest pain and dyspnoe, respectively) (table 2 and online supplemental table S8). Palpitations were least frequent in IHD and most reported in structural non-IHD, primary electrical disease, and SUDS. Near-syncope or syncope occurred with similar frequency across all subgroups of cardiac aetiologies. Survivors with primary electrical disturbances (n=3) generally reported the highest number of symptoms.

The total number of angina equivalent symptoms was more prevalent in patients with structural non-IHDs compared with those with IHD, primary electrical disease and SUDS, although the difference was not statistically significant (p=0.111 and ES=0.26).

Among the 27 patients with no identifiable cause, designated SUDS, genetic testing was reported for 4/17 survivors (23%) and in 5/10 deceased (nine with available autopsy reports).

### Pre-existing comorbidity and CVD risk factors

Prior to OHCA, 15/134 (11%) patients had been diagnosed with one or more CVD (table 1). CVD risk factors were documented in 79/107 (74%) patients with available medical record, with positive family history and overweight as the most frequent. Non-cardiac comorbidities were documented in nearly half of the medical records (n=50). Only 16 (15%) were previously healthy, with no CVD risk factors or other diagnoses.

We did not detect any association between exercise volume and any of these variables, except for the absence of diabetes mellitus in the high-exercise group.

### Pre-existing CVD and CVD risk factors related to the aetiology of OHCA

We did not find any significant differences in pre-existing CVD or CVD risk factors between the different OHCA aetiologies. CVD diagnosed prior to OHCA was determined as the cause of OHCA in eight (four IHD, three cardiomyopathy and one long QT syndrome) out of 15 patients with pre-OHCA CVD diagnosis.

## Discussion

This multiple source study of 134 young Norwegians with OHCA is the first to combine data on pre-event symptoms, circumstances surrounding symptoms, CVD risk factors and exercise volume related to OHCA aetiology in both survivors and deceased of both genders. The main finding was that preceding CVD symptoms were present both during rest/everyday activity and exercise in most victims, reported by more survivors than next-of-kin of the deceased (78% vs 60%). Overall, IHD was the leading cause of OHCA (58% in males vs 38% in females), and 74% of the patients had risk factors for CVD, with positive family history and overweight most frequently reported. SUDS was the most prevalent cause in the youngest (≤35 years). There were no significant differences between exercise habits prior to OHCA and aetiology, symptoms, risk factors or pre-existing CVD.

### Aetiology of OHCA

The prevalence of IHD was comparable to or slightly higher than previously reported, both for the overall population^8 18 19^ and for individuals aged ≤35 years.^20^,^22^ In line with established knowledge, IHD was more prevalent in individuals aged >35 years, compared with those aged ≤35 years.^3^

Studies focusing on individuals younger than 35 years frequently report SUDS as the most prevalent cause of out-of-hospital cardiac death (31%–53%).^20^,^23^ SUDS constitute structurally normal hearts at autopsy, but are presumed to result from an underlying primary electrical disorder or an early stage of non-ischaemic structural heart disease.^24^ Despite 60% of our study population being OHCA survivors and having been through proper clinical examinations after the event, SUDS remained the most common aetiology of OHCA in individuals aged ≤35 years. This underscores the challenge of preventing OHCA through screening. Genetic testing and family screening have the potential to identify a cardiac aetiology in 33%–60% of patients designated as SUDS postautopsy.^25 26^ Sadly then, less than one-quarter of SUDS survivors in our study had a record of genetic testing in their medical record. And in several of the patients, the genetic analysis was limited to mutations associated with long QT syndrome, whereas a wider range of CVD, including cardiomyopathies, is now available for genetic testing. It is likely that the proportion classified as SUDS could decrease with a more extensive use of genetic testing in both OHCA survivors and deceased. This would also provide family members an opportunity to detect potential life-threatening CVD at an early stage and take actions to prevent OHCA.

A recent meta-analysis by D’Ascenzi *et al* found a structurally normal heart as the most common aetiology of sudden cardiac death among both young athletes and non-athletes (≤35 years), with varying prevalence of specific structural cardiac abnormalities.^27^ In Norway, this may be limited by knowledge and experience among coroners in small hospitals. They might not know what to look for in young healthy individuals suffering from OHCA, and there are no national guidelines for cardiac autopsies in athletes. Other countries, like Italy and Great Britain, perform cardiac autopsies of unexpected deaths in regional or national forensic centres, where they can gather experience with rare diagnoses. We found a trend towards a higher IHD prevalence in non-regular exercisers, and most SUDS in those exercising ≥5 hours per week. Still, as emphasised by D’Ascenzi *et al*,^27^ genetic and congenital abnormalities that may trigger OHCA are prevalent across all groups of victims, regardless of exercise volume. This insight should guide OHCA prevention strategies in general.

### Symptoms

The prevalence of CVD symptoms reported by the OHCA survivors was higher than or comparable to previous reports from general population studies in the young (ranging from 54% to 78%).^38 28^,^30^ In sports-related OHCA, previous reports show a lower prevalence (14%–48%) compared with our study.^4^ Common for these studies was that they only included deceased individuals, while we contribute with more knowledge, obtaining data from several sources including survivor voices, who probably have recalled several times what they experienced prior to the OHCA.

The high prevalence of symptoms prior to OHCA is noteworthy, as it represents an opportunity to detect occult CVD and possibly prevent serious disease leading to OHCA.^31 32^ However, the clinical value is complicated by high false positive rates of these symptoms in screening, ranging from 1% to 17% in US junior athletes and school children to 12%–17% in Norwegian Olympic athletes (<50 years).^33 34^ The low prevalence of CVD in these studies results in low specificity and predictive value for CVD symptoms. As the positive predictive value of any symptom or risk factor is influenced by disease prevalence, a risk-based approach to screening could be a reasonable approach.^35^ One such approach would be to assume that patients with CVD symptoms as well as non-specific symptoms like exhaustion, dizziness or reduced physical capacity had a higher risk of OHCA. We found no convincing evidence of that, but that does not rule out such a connection. Many of the survivors who participated in a qualitative study in this project reported fatigue prior to the event, but most did not relate it to CVD at the time,^36^ rather “there were so many reasons for me being tired and exhausted”. Nevertheless, half of the patients had sought healthcare for symptoms prior to OHCA, consistent with previous studies from Sweden and Denmark.^3 29 37^ This observation supports findings from research indicating that many patients had experienced concerns before OHCA.^38^ With targeted examinations, aimed to rule out CVD even in young healthy individuals, some of the diseases may have been detected and OHCA potentially prevented.^3^

### Circumstances of symptoms

In preparticipation health evaluations, it is common to ask for symptoms only during exercise.^13 39 40^ However, a substantial proportion of the patients reported symptoms associated with CVD only during rest/everyday activity. Since all ended up with OHCA, it might be important to ask for CVD symptoms also unrelated to exercise, especially in those who do not exercise regularly. Paratz *et al* interestingly tried to contextualise circumstances to analyse whether more OHCA occurred during sleep, sedentary state or exercise than would be expected based on national time-use data.^8^ Seen in this context, it is not surprising that non-regular exercisers had significantly more symptoms only in rest.

### Symptoms related to aetiology

The distribution of symptoms preceding SCD is highly related to the aetiology of OHCA.^3^ Our data confirmed chest pain as the most prevalent symptom reported by patients with IHD,^28 29 41^ but IHD survivors reported a higher prevalence of dyspnoea (55%) than in previous reports (3%–18%) in deceased individuals. A high burden of symptoms in structural non-IHD, with HCM as the biggest subgroup, aligns with reports from Danish patients with HCM.^37^

Patients classified with SUDS had the lowest number of symptoms, with no symptoms reported by more than one-third of survivors and half of the deceased. That patients with SUDS reported the least symptoms was expected, but the proportion was still higher than reported in studies from the UK and Denmark, which found symptoms in 10%–35% of cases.^42 43^ The higher prevalence of symptoms in our study might be due to the multiple source approach, including data from questionnaires, medical records and both survivors and next-of-kin of the deceased, compared with coroner reports and autopsy data in other studies. Given that SUDS presumably often is caused by undiagnosed arrhythmic disorders, it is notable that the small group with confirmed primary electrical disease had the highest number of symptoms before OHCA, suggesting a potential progression of disease in these patients. Further research to understand the spectrum of diseases currently diagnosed as SUDS is crucial for preventing OHCA in the young.

### Pre-existing comorbidity and risk factors for CVD

The prevalence of recognised CVD prior to OHCA was low (11%), similar to an Australian registry study of individuals aged 1–50 years.^8^ Recent studies report a high prevalence of modifiable risk factors like overweight/obesity, arterial hypertension, hypercholesterolaemia and diabetes mellitus (34%–65%) in young OHCA victims.^8 18 23^

We did not ask about weight in the questionnaire, so if body build was not mentioned in the medical records or autopsy reports, we had to assume it was normal, potentially underestimating the prevalence of overweight (online supplemental table S9). Despite this, overweight emerged as the second most frequent risk factor. The role of obesity as either a risk factor or direct cause of SCD remains debated across studies^44^; nevertheless, the increased risk of OHCA along with rising BMI is well-established.^45^ And as this is also a modifiable risk factor, it is time to focus more on weight-reducing strategies to prevent OHCA.

However, the most striking finding was perhaps that more than half of the patients had a positive family history of CVD, OHCA, arterial hypertension, hypercholesterolaemia or diabetes mellitus. This underlines the importance of performing a thorough medical history to find those who might be at increased risk and with modifiable risk factors.

### Risk factors of CVD related to the aetiology of OHCA

We found no significant association between CVD risk profiles prior to OHCA and its aetiology. However, in half of the patients with prior CVD diagnosis, their diagnosis coincided with the event cause, aligning with data from an Australian registry study.^8^ For these patients, OHCA might have been preventable with appropriate treatment and risk stratification.^46^

### Limitations

There are several limitations to our study. Although we included our study population from the national cardiac arrest registry, NorCAR, the study population may be subject to selection bias, as we could only include patients with consent. It is possible that survivors or next-of-kins who were more aware of CVD risk factors or symptoms were more likely to participate. Having experienced an OHCA represents an obvious selection bias, and our study did not include a control group from the general population. Additionally, the thresholds selected for defining groups based on exercise volume were pragmatically and arbitrarily chosen.

The retrospective assessment of risk factors and symptoms introduces potential recall bias, although this could go either way. The possible underestimation of overweight has been previously discussed. Regarding aetiology, individuals who report CVD symptoms may be more likely to receive a diagnosis of cardiac OHCA compared with asymptomatic patients.

Furthermore, the data presented in this study are between 7 and 10 years old. Given the rapid advancements in cardiovascular genetics, it is likely that both the proportion of cases undergoing genetic investigations and the diagnostic yield would be higher in more recent datasets. Lastly, the sample size for groups stratified by aetiology and exercise volume was small, suggesting a risk of type II statistical errors. ES analysis indicates a moderate to large difference in symptomatology among various OHCA aetiologies. Most comparisons of aetiology and symptoms across pre-OHCA exercise volume showed low ES and low potential for type II errors. However, moderate ESs were observed for symptoms experienced exclusively during rest/everyday activity or during exercise only.

## Conclusions

Preceding symptoms during all circumstances and CVD risk factors were prevalent in young Norwegians who suffered OHCA regardless of exercise volume. Overall, IHD was the leading cause of OHCA, and at least one-third of the patients were overweight. SUDS was most common in the youngest (≤35 years), although increased use of genetic testing might have explained more of these cases and revealed a potential for prevention. We suggest carefully evaluating symptoms, addressing risk factors and increasing the use of genetic testing to help prevent OHCA in young Norwegians, regardless of exercise habits.

## Supplementary material

10.1136/bmjsem-2025-002505 Supplementary file 1

## Data Availability

Data are available on reasonable request.
