# Supplementary material for: Out-of-hospital cardiac arrest in Norwegians aged 12–49 years: nationwide analysis of preceding symptoms and risk factors related to aetiology and pre-arrest exercise habits
Source: BMJ Open Sport Exerc Med. 2025 Jul 1;11(3):e002505. doi: 10.1136/bmjsem-2025-002505 (PMC12215123; doi:10.1136/bmjsem-2025-002505)
Supplement: Supplementary file 1 [file bmjsem-11-3-s001.docx]

Table S1: Questionnaire designed for the study, translated from Norwegian to English

Questionnaire in the event of cardiac arrest in Norway

| **Category** | **Question** | **Free text / Response alternatives** | **Additional information to responder** |
| --- | --- | --- | --- |
| Information about the patient | Number of the study participant | Free text |  |
|  | Country of birth, patient | Free text |  |
|  | Maternal country of birth | Free text |  |
|  | Father's country of birth | Free text |  |
|  | Skin tone, patient | Black / White / Other | The risk of cardiac arrest is affected by ethnicity. In international studies, ethnicity is described by skin tone, and we therefore ask you to state this |
|  | Maternal skin tone | Black / White / Other |  |
|  | Father's skin tone | Black / White / Other |  |
| Previous medical history | For this category, it is possible to tick multiple answers options per question. | | |
|  | Have you experienced any of the following symptoms at rest/during everyday activity? | Chest pain / Abnormal heavy breathing / Fainting / Near-fainting / Palpitations | Examples of everyday activity: Sleeping, eating, walking to the bus, cutting the lawn |
|  | Have you experienced any of the following symptoms during exercise? | Chest pain / Abnormal heavy breathing / Fainting / Near-fainting / Palpitations |  |
|  | Have you been in contact with a health care provider because of these symptoms? | Yes / No / Do not remember / Have not experienced symptoms prior to cardiac arrest |  |
|  | If so, who were you in contact with? | Free text | (Example: GP xxx, Oslo accident and emergency outpatient clinic, St. Olavs Hospital) |
|  | Have you participated in any kind of cardiac screening before participating in sports? | Yes / No / Do not remember | Screening means that you are offered an examination because you are part of a group and not because you have symptoms. For example, everyone in the club/national team is summoned to a doctor for a cardiac examination |
|  | If so, where and who arranged it? | Free text |  |
|  | If you've attended a cardiac screening, what advice did you get? | Continue as before / Reduce the level of activity / Do not remember |  |
|  | Have you been treated for cardiac disease before? | Yes / No |  |
|  | If yes, - what disease | Free text |  |
|  | What treatment | Free text |  |
| Disease of parents, siblings or children before the age of 50 | Cardiac disease | Tick the box to confirm |  |
|  | If so, in whom and which cardiac disease | Free text |  |
|  | High blood pressure | Tick the box to confirm |  |
|  | If so, in whom | Free text |  |
|  | High cholesterol |  |  |
|  | If so, in whom | Free text |  |
|  | Diabetes | Tick the box to confirm |  |
|  | If so, in whom | Free text |  |
|  | Sudden or unexpected death | Tick the box to confirm |  |
|  | If so, in whom and what was the cause of death | Free text |  |
|  | Do not have information on the medical history of family members | Tick the box to confirm |  |
| Exercise the last year prior to cardiac arrest | How many hours did you exercise in an average week? | 0 hours / <5 hours / 5-10 hours / >10 hours | Exercise is defined as physical effort where you sweat / achieve increased heart rate |
|  | Were you a member of a sports team? | Yes/No | If you didn't exercise, you can skip the rest of the questions in this category |
|  | If so, which team: | Free text |  |
|  | What training/competition level were you at? | Recreational / Competitive athlete / Top athlete |  |
|  | Name a few (max 3) competitions you participated in the last year before your cardiac arrest: | Free text | Example: Sentrumsløpet, local championship in football, Norwegian championship, World championship |
|  | Type of exercise | **Endurance** (example; running, cycling, rowing, cross-country skiing, handball) / **Strength** (example; powerlifting, handball) / **Technical** (example; judo, dance, handball) | You can tick multiple answers. For example, handball, can be strength, endurance and technique depending on the training session |
|  | What sport/activity did you perform?  Main sport/activity: | Free text |  |
|  | Any other sports/activities you participated in regularly? | Free text |  |
|  | How many years have you been practicing current exercise or activity | years |  |
| Did cardiac arrest occur in connection with exercise or physical activity? | During exercise/activity | Tick the box to confirm |  |
|  | If yes, specify the type of exercise or activity | Free text |  |
|  | During competition | Tick the box to confirm |  |
|  | If yes, specify the type of competition | Free text |  |
|  | After exercise/activity/ competition | Tick the box to confirm |  |
|  | If yes, specify how long after? | hours and minutes |  |
| Do you feel that you have been offered sufficient follow-up after the cardiac arrest? | I have received good follow-up from the hospital after discharge | Tick the box to confirm |  |
|  | I have received good follow-up from my general practitioner | Tick the box to confirm |  |
|  | I was offered rehabilitation | Tick the box to confirm |  |
|  | No, I do not feel that I have received sufficient follow-up | Tick the box to confirm |  |
|  | If applicable; I have received follow-up from the sports community | Sports team/ Local sports community / national sports federation / I did not receive follow up from the sports community |  |
| Questions related to the cardiac arrest and details of the resuscitation | Did the cardiac arrest happen at a sports venue/outdoor venue? | Yes/No | Some of the questions may be difficult to answer, if so, choose the response alternative "do not know” |
|  | Specify the location of cardiac arrest | Free text |  |
|  | Was the cardiac arrest witnessed? | Yes / No / Do not know |  |
|  | Who started cardiopulmonary resuscitation? | “Next of kin” or friend / bystander / On-site healthcare professionals / Emergency personnel / Do not know |  |
|  | If the cardiac arrest occurred in a sports arena or fitness center, was there an available defibrillator? | Yes / No / Do not know |  |
|  | Was a defibrillator attached before the arrival of the ambulance / medical doctor? | Yes / No / Do not know |  |
|  | Did the defibrillator deliver a shock before the arrival of the ambulance? | Yes / No / Do not know |  |
|  | Was guidance given to cardiopulmonary resuscitation by those who responded to the emergency telephone line (113)? | Yes / No / Do not know |  |
|  | Field for additional comments | Free text |  |

Table S2 Overview of the adjudicated etiology of OHCA related to exercise volume prior OHCA, reported for cases with available medical record and/or autopsy report.

|  | No regular exercise | <5 h of exercise / week | ≥5 h of exercise / week | Test used | χ²-value (df) | P-value | Effect size |
| --- | --- | --- | --- | --- | --- | --- | --- |
|  | n (%) | n (%) | n (%) |  |  |  |  |
| Number of patients | 64 | 60 | 34 |  |  |  |  |
|  |  |  |  | FET | N/A | 0.108 | 0.13 |
| Ischemic heart disease | 18 (60) | 30 (50) | 16 (47) |  |  |  |  |
| Structural non-ischemic heart disease | 8 (27) | 16 (27) | 7 (21) |  |  |  |  |
| Primary electrical disease | 1 (3.3) | 1 (1.7) | 2 (5.9) |  |  |  |  |
| Sudden unexpected death syndrome | 3 (10) | 13 (22) | 9 (27) |  |  |  |  |
| *Hypothesis testing of between group differences is calculated with Fischer's exact test if any expected value is <5. Effect size is presented by Cramer’s V. Abbreviations: χ² (Chi-square test), FET (Fisher's Exact Test), N/A (Not applicable). Abbreviations: χ² (Chi-square test), FET (Fisher's Exact Test), N/A (Not applicable). | | | | | | | |

Table S3 - Symptoms experienced prior to OHCA as retrospectively reported in questionnaire or medical record, and as described in medical records preceding the event. Symptoms associated with cardiac disease is presented for groups divided by exercise-volume prior to OHCA. Table S3 a present data for survivors and S3 b present data for the deceased.

1. Survivors:

|  | No regular exercise | <5 h of exercise/week | ≥5 h of exercise/week |  |  |  |  |
| --- | --- | --- | --- | --- | --- | --- | --- |
|  | n (%) | n (%) | n (%) | Test used | χ²-value (df) | P-value | Effect size |
| Survivors at risk | 15 | 39 | 22 |  |  |  |  |
| Symptoms associated with CVD |  |  |  |  |  |  |  |
| Chest Pain | 9 (60) | 21 (54) | 10 (45) | χ² | 0.80 (2) | 0.669 | 0.10 |
| Dyspnoe | 8 (53) | 20 (51) | 9 (41) | χ² | 0.77 (2) | 0.681 | 0.10 |
| Palpitations | 6 (40) | 15 (38) | 6 (27) | χ² | 0.93 (2) | 0.627 | 0.11 |
| Near syncope/syncope | 4 (27) | 12 (31) | 7 (32) | FET | N/A | 1.000 | 0.04 |
| Number of symptoms |  |  |  |  |  |  |  |
| No symptoms reported | 2 (13) | 9 (23) | 6 (27) | FET | N/A | 0.889 | 0.16 |
| One | 5 (33) | 7 (18) | 7 (32) |  |  |  |  |
| Two | 4 (27) | 12 (31) | 4 (18) |  |  |  |  |
| Three | 2 (13) | 7 (18) | 3 (14) |  |  |  |  |
| Four | 2 (13) | 4 (10) | 2 (9.1) |  |  |  |  |
| *Hypothesis testing of between group differences is calculated with Fischer's exact test if any expected value is <5. Effect size is presented by Cramer’s V. Abbreviations: CVD (Cardiovascular disease), χ² (Chi-square test), FET (Fisher's Exact Test), N/A (Not applicable). | | | | | | | |

1. Deceased:

|  | No regular exercise | <5 h of exercise/week | ≥5 h of exercise/week |  |  |  |  |
| --- | --- | --- | --- | --- | --- | --- | --- |
|  | n (%) | n (%) | n (%) | Test used | χ²-value (df) | P-value | Effect size |
| Deceased at risk | 15 | 21 | 12 |  |  |  |  |
| Symptoms associated with CVD |  |  |  |  |  |  |  |
| Chest Pain | 4 (27) | 5 (24) | 4 (33) | FET | N/A | 0.916 | 0.09 |
| Dyspnoe | 6 (40) | 4 (19) | 3 (25) | FET | N/A | 0.422 | 0.20 |
| Palpitations | 2 (13) | 5 (24) | 2 (17) | FET | N/A | 0.893 | 0.12 |
| Near syncope/syncope | 6 (40) | 4 (19) | 4 (33) | FET | N/A | 0.362 | 0.20 |
| Number of symptoms |  |  |  |  |  |  |  |
| No symptoms reported | 5 (33) | 10 (48) | 5 (42) | FET | N/A | 0.835 | 0.22 |
| One | 5 (33) | 6 (29) | 3 (25) |  |  |  |  |
| Two | 3 (20) | 4 (19) | 2 (17) |  |  |  |  |
| Three | 1 (6.7) | 0 | 2 (17) |  |  |  |  |
| Four | 1 (6.7) | 1 (4.8) | 0 |  |  |  |  |
| *Hypothesis testing of between group differences is calculated with Fischer's exact test if any expected value is <5. Effect size is presented by Cramer’s V. Abbreviations: CVD (Cardiovascular disease), χ² (Chi-square test), FET (Fisher's Exact Test), N/A (Not applicable). | | | | | | | |

Table S4 - Detailed information reported by survivors and by next-of-kin on behalf of deceased on whether the symptoms had been experienced:

1. only during rest /everyday activity
2. during exercise
3. both during rest / everyday activity and during exercise only

The denominator in this equation were the number of respondents who reported pre-OHCA exercise-volume and reported that they had experienced symptoms in the questionnaire. The information is presented for groups divided by pre-OHCA exercise-volume and separately for the a) survivors b) deceased.

1. Survivors:

| Source of data: Questionnaire | Circumstances of symptoms reported by survivors | | | |  |  |  |
| --- | --- | --- | --- | --- | --- | --- | --- |
|  | Non-regular exercisers | <5 h of exercise / week | ≥5 h of exercise / week |  |  |  |  |
|  | n (%) | n (%) | n (%) | Test used | χ²-value (df) | P-value | Effect size |
| Patients with symptoms | 8 | 25 | 12 |  |  |  |  |
| I: Rest / everyday activity, only | 4 (50) | 7 (28) | 2 (17) | FET | N/A | 0.290 | 0.24 |
| II: Exercise, only | 0 | 2 (8) | 3 (25) | FET | N/A | 0.276 | 0.28 |
| III: Both; rest / everyday activity and exercise | 4 (50) | 16 (64) | 7 (58) | FET | N/A | 0.844 | 0.11 |
| *Hypothesis testing of between group differences is calculated with Fischer's exact test if any expected value is <5. Effect size is presented by Cramer’s V. Abbreviations: χ² (Chi-square test), FET (Fisher's Exact Test), N/A (Not applicable). | | | | | | | |

1. Deceased:

| Source of data: Questionnaire | Circumstances of symptoms reported by deceased | | | |  |  |  |
| --- | --- | --- | --- | --- | --- | --- | --- |
|  | Non-regular exercisers | <5 h of exercise / week | ≥5 h of exercise / week |  |  |  |  |
|  | n (%) | n (%) | n (%) | Test used | χ²-value (df) | P-value | Effect size |
| Persons with symptoms | 9 | 9 | 5 |  |  |  |  |
| I: Rest / everyday activity, only | 6 (67) | 6 (67) | 0 | FET | N/A | 0.036 | 0.55 |
| II: Exercise, only | 0 | 1 (11) | 2 (40) | FET | N/A | 0.202 | 0.45 |
| III: Both; rest / everyday activity and exercise | 3 (33) | 2 (22) | 3 (60) | FET | N/A | 0.455 | 0.30 |
| *Hypothesis testing of between group differences is calculated with Fischer's exact test if any expected value is <5. Effect size is presented by Cramer’s V. Abbreviations: χ² (Chi-square test), FET (Fisher's Exact Test), N/A (Not applicable). | | | | | | | |

Table S5 – Post hoc analysis of statistical significantly results from hypothesis testing of differences in the prevalence symptoms occurring at rest / everyday activity, only, analysed by pre-OHCA exercise volume among deceased.

| Symptoms reported by deceased | Rest / everyday activity, only | | | |
| --- | --- | --- | --- | --- |
|  | Test used | χ²-value (df) | P-value^BF^ | Effect size |
| Groups for pair wise comparison |  |  |  |  |
| Non-regular exercisers vs <5 h of exercise / week | FET | N/A | 1.000 | 0.00 |
| Non-regular exercisers vs ≥5 h of exercise / week | FET | N/A | 0.028 | 0.65 |
| 5 h of exercise / week vs ≥5 h of exercise / week | FET | N/A | 0.028 | 0.65 |
| Pairwise comparison is performed trough Fisher's Exact Test if any expected value is <5. Effect size is presented by Cramers' V. Abbreviations: χ² (Chi-square test), FET (Fisher's Exact Test), N/A (Not applicable). ^BF^Alpha-level for significance is adjusted with Bonferroni correction due to multiple testing, ⍺-values <0.01667 are considered statistically significant. | | | | |

Table S6 – Presentation of non-specific symptoms extracted from the medical records by pre-OHCA exercise volume. The table present the prevalence of each reported symptom, as well as the overall prevalence of any angina equivalent symptom, and the number of angina equivalent symptoms.

| Data source |  | | |  |  |  |  |
| --- | --- | --- | --- | --- | --- | --- | --- |
|  | No regular exercise | <5 h of exercise/week | ≥5 h of exercise/week |  |  |  |  |
|  | n (%) | n (%) | n (%) | Test used | χ²-value (df) | P-value | Effect size |
| Number of patients | 22 | 50 | 28 |  |  |  |  |
| Non-specific symptoms |  |  |  |  |  |  |  |
| Generally exhausted^A^ | 7 (32) | 8 (16) | 7 (25) | FET | N/A | 0.287 | 0.16 |
| Dizziness^A^ | 3 (14) | 9 (18) | 5 (18) | FET | N/A | 0.942 | 0.05 |
| Reduced physical capacity^A^ | 0 | 4 (8.0) | 3 (11) | FET | N/A | 0.350 | 0.15 |
| Nausea^A^ | 3 (14) | 2 (4.0) | 1 (3.6) | FET | N/A | 0.253 | 0.17 |
| Stomach pain^A^ | 0 | 3 (6.0) | 1 (3.6) | FET | N/A | 0.808 | 0.12 |
| Shoulder-/neck-/back-pain | 5 (23) | 5 (10) | 5 (18) | FET | N/A | 0.331 | 0.15 |
| Paraesthesia/weakness in arms | 1 (4.5) | 3 (6.0) | 2 (7.1) | FET | N/A | 1.000 | 0.04 |
| Jaw pain | 0 | 1 (2.0) | 1 (3.6) | FET | N/A | 1.000 | 0.09 |
| Symptoms associated with infectious disease last days | 2 (9.1) | 4 (8.0)) | 2 (7.1) | FET | N/A | 1.000 | 0.03 |
| Any angina equivalent symptom | 9 (41) | 15 (30) | 12 (43) | χ² | 1.58 (2) | 0.453 | 0.13 |
| Number of angina equivalent symptoms |  |  |  | FET | N/A | 0.579 | 0.14 |
| None reported | 13 (59) | 35 (70) | 16 (57) |  |  |  |  |
| One | 6 (27) | 6 (12) | 8 (29) |  |  |  |  |
| Two | 2 (9.1) | 7 (14) | 3 (11) |  |  |  |  |
| Three | 1 (4.5) | 2 (4.0) | 1 (3.6) |  |  |  |  |
| ^A^ Angina equivalent symptoms. Hypothesis testing of between group differences is calculated by Fischer's exact test if any expected value is <5. Effect size is presented by Cramer’s V. Abbreviations: χ² (Chi-square test), FET (Fisher's Exact Test), N/A (Not applicable). | | | | | | | |

Table S7 - Symptoms experienced prior to OHCA as retrospectively reported in questionnaire or medical record, and as described in medical records preceding the event. Symptoms associated with cardiac disease is presented for groups divided by gender. Table S5 a present data for survivors and S5 b present data for the deceased.

a)

|  | Females | Males |  |  |  |  |
| --- | --- | --- | --- | --- | --- | --- |
|  | n (%) | n (%) | Test used | χ²-value (df) | P-value | Effect size |
| Survivors at risk | 21 | 60 |  |  |  |  |
| Symptoms associated with CVD |  |  |  |  |  |  |
| Chest Pain | 12 (57) | 31 (52) | χ² | 0.19 (1) | 0.665 | 0.05 |
| Dyspnoe | 11 (52) | 29 (48) | χ² | 0.10 (1) | 0.749 | 0.04 |
| Palpitations | 11 (52) | 18 (30) | χ² | 3.39 (1) | 0.066 | 0.21 |
| Near syncope/syncope | 5 (24) | 18 (30) | χ² | 0.29 (1) | 0.588 | 0.06 |
| Number of symptoms |  |  | FET | N/A | 0.547 | 0.19 |
| No symptoms reported | 2 (9.5) | 16 (27) |  |  |  |  |
| One | 7 (33) | 14 (23) |  |  |  |  |
| Two | 6 (29) | 14 (23) |  |  |  |  |
| Three | 4 (19) | 10 (17) |  |  |  |  |
| Four | 2 (9.5) | 6 (10) |  |  |  |  |
| Hypothesis testing of between group differences is calculated by Fischer's exact test if any expected value is <5. Effect size is presented by Cramer’s V. Abbreviations: CVD (Cardiovascular disease), χ² (Chi-square test), FET (Fisher's Exact Test), N/A (Not applicable). | | | | | | |

b)

|  | Females | Males |  |  |  |  |
| --- | --- | --- | --- | --- | --- | --- |
|  | n (%) | n (%) | Test used | χ²-value (df) | P-value | Effect size |
| Deceased at risk | 16 | 37 |  |  |  |  |
| Symptoms associated with CVD |  |  |  |  |  |  |
| Chest Pain | 4 (25) | 11 (30) | FET | N/A | 1.000 | 0.05 |
| Dyspnoe | 7 (44) | 8 (22) | FET | N/A | 0.182 | 0.23 |
| Palpitations | 4 (25) | 5 (14) | FET | N/A | 0.427 | 0.14 |
| Near syncope/syncope | 6 (38) | 8 (22) | FET | N/A | 0.311 | 0.17 |
| Number of symptoms |  |  | FET | N/A | 0.269 | 0.32 |
| No symptoms reported | 5 (31) | 16 (43) |  |  |  |  |
| One | 6 (38) | 12 (32) |  |  |  |  |
| Two | 2 (13) | 7 (19) |  |  |  |  |
| Three | 1 (6.3) | 2 (5.4) |  |  |  |  |
| Four | 2 (13) | 0 |  |  |  |  |
| Hypothesis testing of between group differences is calculated by Fischer's exact test if any expected value is <5. Effect size is presented by Cramer’s V. Abbreviations: CVD (Cardiovascular disease), χ² (Chi-square test), FET (Fisher's Exact Test), N/A (Not applicable). | | | | | | |

Table S8 – Post hoc analysis of statistical significantly results from hypothesis testing of differences in the prevalence of CVD symptoms reported for the different subgroups of cardiac OHCA. Results are presented separately for survivors and deceased.

1. Survivors

| Symptoms reported by survivors | Chest pain | | | | Dyspnoe | | | |
| --- | --- | --- | --- | --- | --- | --- | --- | --- |
|  | Test used | χ²-value (df) | P-value^BF^ | Effect size | Test used | χ²-value (df) | P-value^BF^ | Effect size |
| Pair comparison |  |  |  |  |  |  |  |  |
| Ischemic heart disease vs Structural Non-Ischemic heart disease | χ² | 1.17 (1) | 0.28 | 0.14 | χ² | 0.02 (1) | 0.904 | 0.02 |
| Ischemic heart disease vs Primary electrical disease | FET | N/A | 0.542 | 0.17 | FET | N/A | 0.254 | 0.22 |
| Ischemic heart disease vs Sudden unexpected death syndrome | χ² | 14.68 (1) | <0.001 | 0.48 | χ² | 7.15 (1) | 0.007 | 0.33 |
| Structural Non-Ischemic heart disease vs Primary electrical disease | FET | N/A | 0.228 | 0.39 | FET | N/A | 0.515 | 0.34 |
| Structural Non-Ischemic heart disease vs Sudden unexpected death syndrome | FET | N/A | 0.044 | 0.42 | FET | N/A | 0.03 | 0.41 |
| Primary electrical disease vs Sudden unexpected death syndrome | FET | N/A | 0.009 | 0.73 | FET | N/A | 0.018 | 0.64 |
| Pairwise comparison is performed trough Fisher's Exact Test if any expected value is <5. Effect size is presented by Cramer’s V. Abbreviations: χ² (Chi-square test), FET (Fisher's Exact Test), N/A (Not applicable). ^BF^Alpha-level for significance is adjusted with Bonferroni correction due to multiple testing, ⍺-values <0.0083 are considered statistically significant. | | | | | | | | |

1. Deceased

| Symptoms reported by deceased | Chest pain | | | |
| --- | --- | --- | --- | --- |
|  | Test used | χ²-value (df) | P-value^BF^ | Effect size |
| Pair comparison |  |  |  |  |
| Ischemic heart disease vs Structural Non-Ischemic heart disease | χ² | 12.3 (1) | <0.001 | 0.54 |
| Ischemic heart diseas vs Primary electrical disease | FET | N/A | 0.458 | 0.227 |
| Ischemic heart disease vs Sudden unexpected death syndrome | FET | N/A | 0.021 | 0.43 |
| Structural Non-Ischemic heart disease vs Primary electrical disease | FET | N/A | 1.000 | 0.05 |
| Structural Non-Ischemic heart disease vs Sudden unexpected death syndrome | FET | N/A | 1.000 | 0.09 |
| Primary electrical disease vs Sudden unexpected death syndrome | FET | N/A | 1.000 | 0.10 |
| Pairwise comparison is performed trough Fisher's Exact Test if any expected value is <5. Effect size is presented by Cramer's V. Abbreviations: χ² (Chi-square test), FET (Fisher's Exact Test), N/A (Not applicable). ^BF^Alpha-level for significance is adjusted with Bonferroni correction due to multiple testing, ⍺-values <0.0083 are considered statistically significant. | | | | |

Table S9 – Overview of available data from medical records and autopsy reports for the variable “overweight”.

| Data source | Normal weight | Overweight | Total* | Missing |
| --- | --- | --- | --- | --- |
| Data source | n (%) | n (%) |  |  |
| Medical record BMI or H&W | 7 (39) | 11 (61) | 18 | 89 |
| Medical record Body built | 19 (51) | 18 (49) | 37 | 70 |
| Autopsy report H&W | 9 (26) | 25 (74) | 34 | 3 |
| All sources combined | 32 (41) | 46 (59) | 78 | 56 |
| All sources combined, missing equals normal weight | 32 (24) | 102 (76) | 134 | N/A |
| *Total number of cases with information on body weight and height / BMI or body build from medical record or autopsy report. Abbreviations: BMI (Body Mass Index), H&W (Height & Weight). | | | | |

Figure S1 – Flowchart of the inclusion, beginning with the population available for inclusion from NorCAR


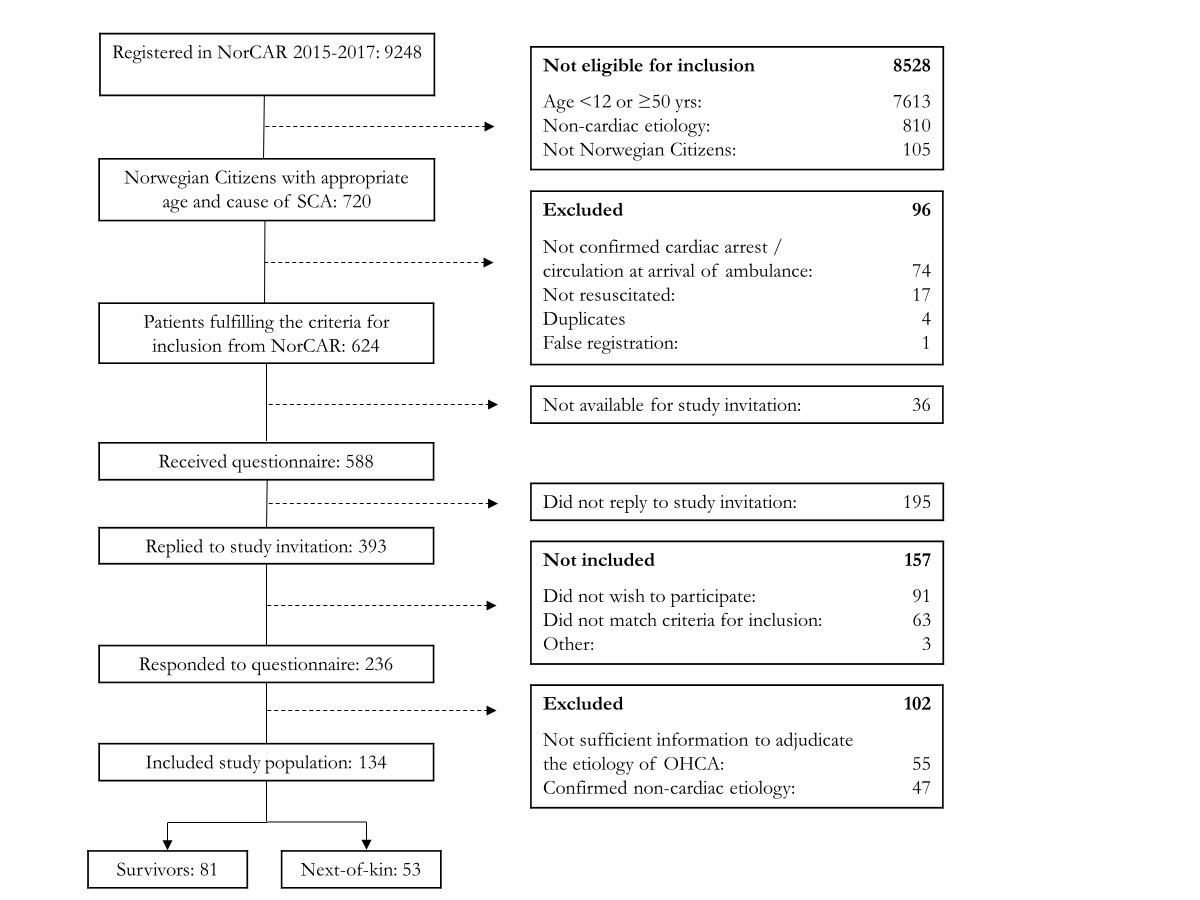


Figure S2 – Etiology of OHCA presented for a) females and b) males.

a)

b)

Figure S3 – Etiology of OHCA presented for a) age ≤35 years at time of OHCA and b) age >35 years at time of OHCA.
